# Use of Gain-of-Function Screening to Identify miRNAs Involved in Paclitaxel Resistance in Triple-Negative Breast Cancer

**DOI:** 10.3390/ijms252413630

**Published:** 2024-12-20

**Authors:** Stéphane Nemours, Carla Solé, Ibai Goicoechea, María Armesto, María Arestin, Ander Urruticoechea, Marta Rezola, Isabel Álvarez López, Roel Schaapveld, Iman Schultz, Lei Zhang, Charles H. Lawrie

**Affiliations:** 1 Molecular Oncology Group, Biogipuzkoa Health Research Institute, 20014 San Sebastian, Spain; stephane.nemours@bio-gipuzkoa.eus (S.N.); carla.solecanadas@bio-gipuzkoa.eus (C.S.); maria.armestoalvarez@bio-gipuzkoa.eus (M.A.); maria.arestinmuruzabal@bio-gipuzkoa.eus (M.A.); 2 Department of Personalized Medicine, NASERTIC, Government of Navarra, 31011 Pamplona, Spain; 3 Breast Cancer Group, Biogipuzkoa Health Research Institute, 20014 San Sebastian, Spain; aurruticoechea@onkologikoa.org (A.U.); isabelmanuela.alvarezlopez@osakidetza.eus (I.Á.L.); 4 Gipuzkoa Cancer Unit, OSI Donostialdea—Onkologikoa Foundation, Paseo Dr Begiristain 121, 20014 San Sebastian, Spain; 5 Department of Pathology, Hospital Universitario Donostia Osakidetza, 20014 Donostia, Spain; marta.rezolabajineta@osakidetza.eus; 6 InteRNA Technologies, 3584 Utrecht, The Netherlands; schaapveld@interna-technologies.com (R.S.); schultz@interna-technologies.com (I.S.); 7 Sino-Swiss Institute of Advanced Technology (SSIAT), Shanghai University, Shanghai 201800, China; zhangleich@shu.edu.cn; 8 IKERBASQUE, Basque Foundation for Science, 48011 Bilbao, Spain; 9 Radcliffe Department of Medicine, University of Oxford, Oxford OX1 2JD, UK

**Keywords:** triple-negative breast cancer, paclitaxel, microRNA, functional screen

## Abstract

Paclitaxel is a widely used chemotherapeutic agent for the treatment of breast cancer (BC), including as a front-line treatment for triple-negative breast cancer (TNBC) patients. However, resistance to paclitaxel remains one of the major causes of death associated with treatment failure. Multiple studies have demonstrated that miRNAs play a role in paclitaxel resistance and are associated with both disease progression and metastasis. In the present study, we used a miRNA-encoding lentiviral library as a gain-of-function screen for paclitaxel resistance in the MDA-MB-231 TNBC cell line. We identified that *miR-181b*, *miR-29a*, *miR-30c*, *miR-196* and *miR-1295* conferred a resistant phenotype to cells. The expression of *miR-29a* also induced resistance to eribulin and vinorelbine, while *miR-181b* and *miR-30c* induced resistance to vinorelbine. We measured the levels of these miRNAs in breast cancer patients and observed higher levels of *miR-29a* in treatment-refractory patients. Taken together, we suggest that *miR-29a* and *miR-181b* may be good candidates for miRNA inhibition to overcome resistance to chemotherapy.

## 1. Introduction

Breast cancer (BC) is a heterogeneous disease consisting of several different subtypes that, until recently, was classified according to the presence of estrogen receptor (ER), progesterone receptor (PR) and/or human epidermal growth factor receptor 2 (HER2) hormone receptors [1,2,3]. Triple-negative breast cancer (TNBC), accounting for 10–17% of BC patients [4], lacks any of these receptors and is the most aggressive form of BC with the worst prognostic outcome [5,6,7]. As TNBC patients do not have drug-targetable receptors, patients typically receive conventional neoadjuvant chemotherapies, particularly anthracycline/taxane combinations. TNBC patients who achieve a complete response after conventional treatment modalities have similar survival prospects to other BC subtypes [8,9]. Paclitaxel, together with anthracyclines, is used as an adjuvant therapy in both primary and refractory BC, particularly in basal-like BC and in BCs that have high-risk indications such as premenopausal; ER-, PR- or HER2-negative; large tumors; or patients with lymph nodes involved [10,11,12]. Paclitaxel is a taxane that targets tubulin, promoting and stabilizing the microtubule assembly during mitosis, thereby activating the spindle assembly checkpoint and generating cell cycle arrest or cell death [13,14,15,16].

Although there is a clear benefit from chemotherapy treatments such as paclitaxel for BC patients, resistance remains a major problem and 10–30% of early-stage BC patients are refractory to treatment [17]. Patients who are either refractory to initial treatment or who undergo a consequent relapse due to innate and/or acquired resistance have worse survival rates [8,9]. This is especially worrying in TNBC patients because of a lack of alternative therapeutic options [18]. At the molecular level, several mechanisms have been proposed to be involved in chemotherapy resistance, including (a) drug transporters and metabolic enzymes that decrease the intracellular drug concentrations; (b) molecular alterations that have a direct impact on cell cycle arrest, apoptosis or DNA repair mechanisms; (c) the promotion of pro-oncogenic signaling pathways; (d) epigenetic alterations such as histone acetylation and DNA methylation; and (e) a reduction in drug target availability [19]. miRNAs have been reported to play key roles in drug resistance for many cancer types, including breast, ovarian, lung, prostate, gastric, renal, colon, hepatocellular carcinoma, cholangiocarcinoma, neuroblastoma and leukemia [19,20,21,22]. In BC, in particular, miRNAs have been described to modulate resistance/sensitivity to several drugs, including radiotherapy, hormone therapy, targeted therapies and chemotherapy [23].

**Figure 1:**
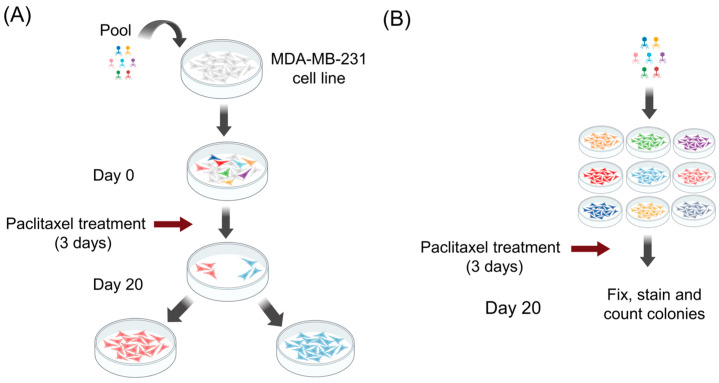
Schematic diagram of (**A**) gain-of-function screening and (**B**) individual clone paclitaxel resistance testing regime. Figure created in parts using BioRender.com.

Despite the widespread use of comparative omic techniques, including microarray and next-generation sequencing (NGS) in biology, these techniques suffer from a large number of indirect false-positives, which makes it challenging to identify the true drivers of phenotypic change. An alternative approach is functional screening, which can more directly identify the genes/RNAs involved in phenotypic changes such as drug resistance. Consequently, we used a lentivirus library encoding 698 human miRNAs as a gain-of-function screen (Figure 1) to identify the miRNAs involved in paclitaxel resistance in TNBC.

## 2. Results

### 2.1. Sensitivity of Breast Cancer Cell Lines to Paclitaxel

Dose–response curves of eleven breast cell lines treated with paclitaxel were obtained to select the best model for these experiments. As can be seen in Table 1, the most sensitive cell line was the TNBC cell line MDA-MB-231, which had a concentration of 50% for the maximal inhibition of cell proliferation (GI_50_) of 0.61 nM compared with an average GI_50_ of 121.03 nM. ER-negative cell lines were significantly more sensitive to paclitaxel than ER-positive cell lines, whereas the sensitivity of PR- and Her2-positive cell lines was similar to PR- and Her2-negative cell lines (Supplementary Figure S1). The TNBC cell lines were also more sensitive to paclitaxel than non-TNBC cells, although not significantly.

### 2.2. Identification of miRNAs Implicated in Paclitaxel Resistance

In order to identify the miRNAs involved in paclitaxel resistance in the MDA-MB-231 cell line, we carried out gain-of-function screening using a lentiviral library encoding 698 miRNAs. Cells were transfected with the library and treated with paclitaxel for 72 h. Subsequently, individual cells were isolated and grown for a further 20 days under antibiotic selection. Thirty-six colonies were generated in this manner and expanded. The resulting clones were tested for resistance to paclitaxel treatment using dose-dependence experiments with MTT assays. Seventeen of the clones demonstrated a significant increase in paclitaxel GI_50_ compared with parental MDA-MB-231 cells (*p* < 0.001) (Supplementary Table S1).

To elucidate the miRNAs encoded by each of these clones, we used vector-specific primers for PCR and visualized the resulting amplicons using electrophoresis (Supplementary Figure S2). The majority of the clones displayed multiple bands, suggesting multiple clones were present and, therefore, we sub-cloned the amplicons into a T/A vector (pCR4-TOPO) and transfected this into bacteria. Clones that displayed single colonies were directly sequenced. Single colonies were picked (>20 colonies per clone) and re-amplified, leading to a total of more than 260 amplicons that were Sanger-sequenced and the detection of 54 different miRNAs (Table 2). Fourteen of these miRNAs were selected on the basis of frequency and relevance to cancer. Most of the miRNAs (11/14) that were selected had at least four sequenced clones and all selected miRNAs were present in at least two publications (in PubMed), suggesting a relationship with either breast cancer and/or drug resistance in cancer. The selected miRNAs were *miR-23a*, *miR-29a*, *miR-30c*, *miR-134*, *miR-155*, *miR-181b*, *miR-196a*, *miR-329*, *miR-337*, *miR-491*, *miR-543*, *miR-650*, *miR-1295* and *miR-let-7a*.

### 2.3. Drug-Resistance Adaptation of Cells Infected with Individual miRNAs

**Figure 2:**
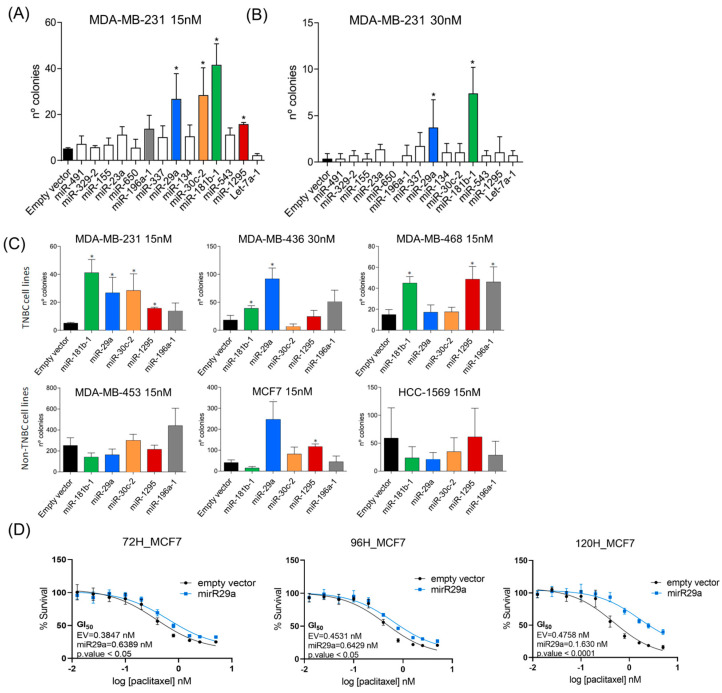
Validation of miRNAs resistant to paclitaxel. Selected miRNAs from functional screening were individually transfected in MDA-MB-231 cells, then submitted to drug treatment. Number of paclitaxel-resistant colonies are represented. (**A**) When cells were treated with 15 nM paclitaxel, *miR-29a*, *miR-30c*, *miR-181b* and *miR-1295* formed significantly more resistant colonies than the empty vector (*p* < 0.05). *miR-196a* also formed more colonies than the control, although this was not significant. (**B**) When cells were treated with 30 nM paclitaxel, *miR-29a* and *miR-181b* formed significantly more resistant colonies than the empty vector (*p* < 0.05). (**C**) Paclitaxel-resistance adaptation experiment was extended to other BC cell lines (TNBC cell lines in the top panel and non-TNBC cell lines in the lower panel were at 15 nM or 30 nM. (**D**) To validate drug-resistance adaptation experiments, MCF7 cells were transduced with a lentivirus miRNA that overexpressed miR-29a. MTT test was performed at 72, 96 and 120 h after treatment with paclitaxel. MCF7 cells overexpressing *miR-29a* showed a higher percentage of survival compared with the empty vector at every timepoint (* = *p* < 0.05).

To validate the resistant phenotype of the identified miRNAs, we transfected MDA-MB-231 cells with lentiviruses encoding individual miRNAs and tested the resultant cells for paclitaxel resistance. Cells infected with *miR-29a*, *miR-30c*, *miR-181b* and *miR-1295* showed a significant increase in the number of colonies compared with cells infected with a control virus when treated with either 15 nM or 30 nM paclitaxel (*p* < 0.05) (Figure 2A,B).

To investigate if resistance was cell-line-specific, we repeated the experiment on MDA-MB-231, MDA-MB-436, MDA-MB-468, MDA-MB-453, MCF-7 and HCC-1569 cells transfected with *miR-181b*-, *miR-29a*-, *miR-30c*-, *miR-1295*- or *miR-196a*-encoding lentiviruses (Figure 2C). The latter virus was included as although it was not significantly resistant (*p* = 0.108), there was an evident trend in that direction. As can be seen from Figure 2C, the overexpression of *miR-181b* significantly increased the resistance to paclitaxel in TNBC cell lines but not non-TNBC cell lines. Cells infected with *miR-29a* induced resistance to paclitaxel in the MDA-MB-231 and MDA-MB-436 TNBC cell lines and also the ER/PR^+^ MCF7 cell line. *miR-30c* appeared to confer resistance only in MDA-MB-231 cells, whereas the overexpression of *miR-1295a* conferred resistance to MDA-MB-231, MDA-MB-468 and MCF7 cells. Although the resistant phenotype of *miR-196a* was not significant in MDA-MB-231 cells (*p* = 0.108), it was significant in TNBC MDA-MB-468 cells (*p* < 0.05) (Figure 2C). The mRNA levels of selected miRNA targets were also measured using qRT-PCR for some cell lines (Supplementary Figure S3). To further confirm the resistant phenotype, dose–response curves were produced at various timepoints for MCF7 cells transduced with *miR-29a.* As can be seen from Figure 2D, cells remained significantly resistant for at least 5 days.

As we were able to produce the resistant phenotype by overexpressing the identified miRNAs in some cell lines but not others, we hypothesized that this could be the result of differences in the endogenous expression of the miRNAs between cell lines or might even explain differences in the basal-resistant phenotype of the different cell lines. Therefore, we measured the levels of *miR-181b*, *miR-29a*, *miR-30c*, *miR-196a* and *miR-1295* in different cell lines using qRT-PCR. We found no significant correlation between the endogenous levels of these miRNAs and the paclitaxel GI_50_ values in the various cell lines (Supplementary Figure S4).

### 2.4. Inhibition of miRNA Expression with LNAs

**Figure 3:**
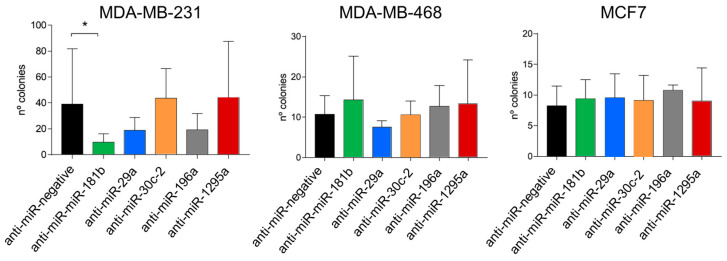
Cell sensitivity to paclitaxel treatment following miRNA inhibition with LNAs. MDA-MB-231, MDA-MB-468 and MCF7 cells were transfected with LNAs against *miR-181b*1, *miR-29a*, *miR-30c*, *miR-196a* and *miR-1295*. Transfected cells were then treated with 15 nM paclitaxel, an adaptation experiment was performed and the number of paclitaxel-resistant colonies was counted. For MDA-MB-231 cells, only *miR-181b* inhibition significantly sensitized cells compared with a negative control. For MDA-MB-468 and MCF7 cells, miRNA inhibition did not sensitize cells upon paclitaxel treatment. Either a *t*-test or Mann–Whitney tests were used for the statistical analysis. Experiments were performed in triplicate (* = *p* < 0.05).

To ascertain if inhibiting the identified miRNAs could reverse the phenotype, thereby rendering cells more sensitive to paclitaxel, we treated the cells with locked nucleic acid (LNA) antagomirs against *miR-181b, miR-29a, miR-30c, miR-196a* and *miR-1295a*. The LNAs were used to transfect MDA-MB-231, MDA-MB-468 and MCF7 cells, which were then treated with paclitaxel. In the case of MDA-MB-231 cells, the inhibition of *miR-181b* significantly increased the sensitivity of the cells to paclitaxel treatment (*p* < 0.05) (Figure 3). The inhibition of *miR-29a* and *miR-196a* also resulted in a sensitization of cells, although not significantly so. In contrast, the inhibition of resistant-associated miRNAs did not significantly sensitize either MDA-MB-468 or MCF7 cells to paclitaxel; however, *miR-29a* inhibition increased the sensitivity of MDA-MB-468 cells, although not significantly so.

### 2.5. miRNA-Driven Resistance Against Paclitaxel-Related Anti-Mitotic Compounds

**Figure 4:**
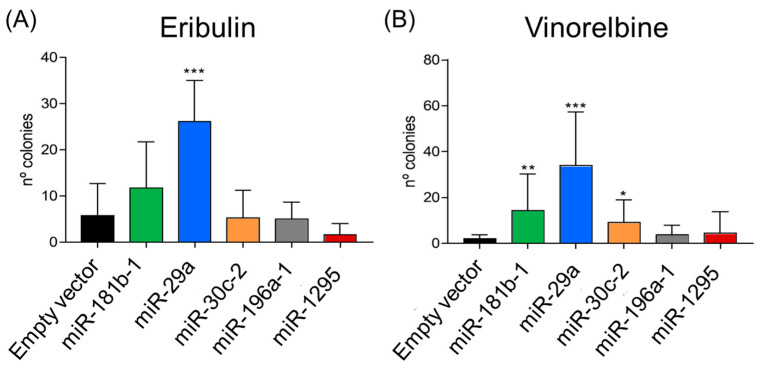
Effect of miRNA overexpression on resistance to eribulin or vinorelbine treatments. Eribulin- and vinorelbine-resistance adaptation experiments were performed using MDA-MB-231 cells overexpressing different miRNAs. (**A**) Number of eribulin-resistant colonies on cells treated with 10 nM eribulin. miR-29a formed significantly more resistant colonies than he empty vector. (**B**) Number of vinorelbine-resistant colonies on cells treated with 75 nM vinorelbine. *miR-181b*, *miR-29a* and *miR-30c* formed significantly more resistant colonies than the empty vector. Either a *t*-test or Mann–Whitney tests were used for the statistical analysis (* = *p* < 0.05; ** = *p* < 0.01; *** = *p* < 0.001).

In order to further investigate paclitaxel resistance, we also tested resistance to eribulin and vinorelbine, anti-mitotic drugs also used to treat breast cancer. We treated MDA-MB-231 cells infected with lentiviral viruses overexpressing *miR-181a*, *miR-29a*, *miR-30c*, *miR-1295* or *miR-196a* with 10 nM eribulin or 75 nM vinorelbine (based on dose–response experiments). *miR-29a*-expressing cells were significantly more resistant than control cells when treated with either eribulin or vinorelbine (*p* < 0.001). The expression of either *miR-181b* or *miR-30c* also conferred resistance to vinorelbine (*p* < 0.01 and *p* < 0.05, respectively) but not to eribulin (Figure 4).

### 2.6. miRNA Expression in a Cohort of Patients Treated with Paclitaxel

**Figure 5:**
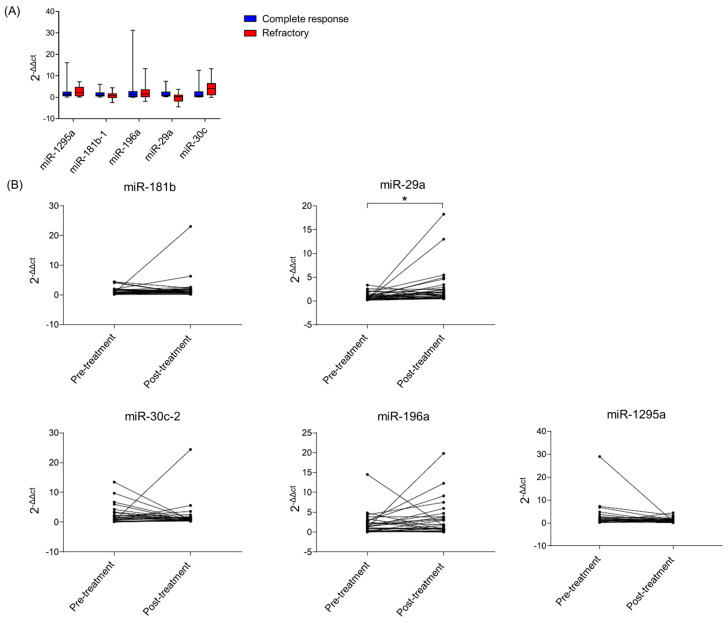
miRNA expression in a cohort of patients treated with paclitaxel. The *miR-181b*, *miR-29a*, *miR-30c*, *miR-196a* and *miR-1295a* expression of tumor samples was evaluated using qRT-PCR assays. (**A**) miRNA expression in pre-treatment samples of patients who achieved a complete response and refractory patients showed no significant difference. (**B**) miRNA expression of refractory patients pre- and post-treatment was compared. Only *miR-29a* expression significantly increased in post-treatment samples compared with pre-treatment samples (* = *p* < 0.05). Either a paired *t*-test or Wilcoxon matched-pairs signed-rank tests were used for the statistical analysis.

In order to investigate if the expression of the identified miRNAs was associated with paclitaxel resistance in clinical BC samples, we measured their expression in 42 BC cases treated with paclitaxel either as a single agent or in combination. The hormone receptor status, histological grade and treatment of patients used in this study can be found in Table 3. The expression levels of *miR-181b*, *miR-29a*, *miR-30c, miR-196a* and *miR-1295a* were measured using pre-treatment samples and were found to be not statistically different between patients who achieved a complete response (CR) and patients who were refractory to therapy (Figure 5A). We compared the expression of these miRNAs between pre- and post-treatment samples with the non-CR patients (*n* = 16) and observed that the expression of *miR-29a* significantly increased in response to treatment (Figure 5B). The other miRNAs did not significantly modify their expression after treatment.

## 3. Discussion

The dysregulation of miRNAs in breast cancer has been reported many times [23,24,25,26,27,28,29,30,31,32,33] and, furthermore, has been demonstrated to play a key role in resistance to treatment in this cancer [33,34,35,36,37,38,39,40]. For example, miRNAs such as *miR-21*, *miR-125b*, *miR-520h* and *miR-18a* have been reported to induce resistance to paclitaxel treatment in BC [41,42,43,44,45,46], whereas *miR-let-7a*, *miR-342*, *miR-34a*, *miR-218*, *miR-100* and *miR-24*, have been reported to sensitize BC tumor cells [47,48,49,50,51]. Although such studies are without doubt valuable, functional screening offers a powerful tool to uncover the molecules associated with a specific phenotype that can be overlooked by traditional comparative omic studies. Furthermore, a gain-of-function approach allows identified molecules to be tested beyond the functional limits of endogenous levels used in omics studies, a useful trait when considering potential clinical usage. In the current study, we used a lentiviral library encoding 698 miRNAs as a gain-of-function screen to identify the miRNAs involved in the resistance of BC cell lines to paclitaxel treatment. As far as we are aware, this is the first study to utilize this strategy to explore drug resistance in breast cancer.

Before carrying out the functional screening, we measured the sensitivity of 11 BC cells lines to paclitaxel treatment. All cell lines were sensitive to this drug, with a wide range of GI_50_ values, and had different sensitivity to paclitaxel, ranging from 0.61 nM (MB-MDA-231 cells) to 1000 nM (ZR51 cells). Interestingly, their sensitivity could be grouped according to the hormone receptor status of the BC, with the TNBC cell lines being the most sensitive, as previously reported [52]. Tabuchi et al. previously reported that ER-negative (150 ng/mL) cell lines were more sensitive to paclitaxel than ER-positive (>500 ng/mL) cell lines [53].

Fifty-four miRNAs were identified from the functional screening and fourteen were selected for validation. *miR-181b*, *miR-29a*, *miR-30c*, *miR-196a* and *miR-1295a* were functionally validated using individual lentiviral infections. In order to explore the resistant phenotype further, we overexpressed the five identified miRNAs in other BC cell lines.

Previous studies have reported that responses to paclitaxel did not differ when comparing BC patients with different hormone-status or HER2-expression diseases [54]. However, *miR-181b* has previously been reported to be overexpressed in TNBC compared with normal tissue [55]. Interestingly, *miR-181b* expression increases in high-grade tumors and in tumors with a high Ki67 expression, demonstrating a correlation with BC aggressiveness [56]. The overexpression of *miR-181b* has also been described to be involved in doxorubicin resistance [57,58] as well as in chemoresistance-related pathways in TNBC [55].

In this study, it was observed that cells infected with *miR-29a* also induced resistance to paclitaxel in TNBC cell lines (MDA-MB-231 and MDA-MB-436) but this phenotype was not subtype-specific as *miR-29a* also induced resistance in the MCF7 cell line (ER^+^). Similar to our results, *miR-29a* overexpression was reported in a paclitaxel-resistant colorectal carcinoma cell line (SW480) [59]. A targeted regulatory relationship between *miR-29a* and PTEN was also observed [59]. BC resistance in cells where *miR-29a* is expressed has been reported for several drugs. Zhong et al. found that cells transfected by *miR-29a* mimics developed Adriamycin and docetaxel resistance in MCF7 cells and were significantly increased compared with control cells [60]. Additionally, cells transfected with *miR-29a* inhibitors could also change the drug resistance of the target cells and restored their sensitivity to Adriamycin and docetaxel to a degree. The authors suggested that *miR-222* and *miR-29a* could regulate the expression of PTEN, possibly the route through which the two miRNAs could confer Adriamycin and docetaxel resistance in MCF7 cells [60].

Cells infected with *miR-30c* were found to increase paclitaxel resistance only in MDA-MB-231 cells. Previously, *miR-30c* has been described as a tumor suppressor to be suppressed in aggressive BC [60], reducing cell proliferation as well as invasion in MDA-MB-231 cells [61].

Cells infected with *miR-196a* formed resistant colonies in the MDA-MB-468 cell line. *miR-196a* overexpression has previously been associated with tumor progression, promoting the growth, differentiation and metastasis of tumor cells [62,63,64,65]. The upregulation of this miRNA in tumor tissues and in plasma correlated with chemoresistance to Adriamycin and paclitaxel in head and neck cancer patients [66]. In BC, *miR-196a* was reported to be regulated by ER and to be a prognostic biomarker for ER-positive BC patients [67]. Cells infected with *miR-1295a* formed resistant colonies in MDA-MB-231, MDA-MB-468 and MCF7 cell lines. To our knowledge, this is the first time that *miR-1295a* has been reported to play a role in BC resistance to paclitaxel. Consistent with the resistant phenotype, when the *miR-181b* expression was inhibited using LNAs, we observed a sensitization of cells to paclitaxel treatment (in MDA-MB-231 cells). Similarly, *miR-29a* inhibition also sensitized MDA-MB-231 and MDA-MB-468 cells, although not significantly. We did not find a reversed phenotype with either an inhibition of *miR-196a* or *miR-1295a*, suggesting that other factors were involved in the resistance mechanisms involving these two miRNAs.

*miR-29a* overexpression also conferred resistance to both vinorelbine and eribulin, also clinically used as anti-mitotic compounds [68]. In contrast, *miR-181b* and *miR-30c* conferred resistance to eribulin but not to vinorelbine. This change in the resistant phenotype probably points to different mechanisms in the miRNAs. Consistent with this idea, although the three compounds are anti-mitotic, they work in different ways as paclitaxel promotes the assembly of microtubules, whereas vinorelbine and eribulin destabilize the microtubule assembly and involve different gene pathways [69]. Interestingly, *miR-29a* has also been reported to be responsible for Adriamycin and docetaxel resistance, suggesting it may function more generically than the other identified miRNAs, for example, by targeting PTEN, as has previously been described [60,70].

In order to examine the expression of the identified miRNAs in a cohort of paclitaxel-treated BC patients, we compared the expression of patients who experienced a complete response (CR) with those who did not respond to treatment. No significant differences in miRNA expression were observed between responder and non-responder patients. We observed that the *miR-29a* expression significantly increased in the post-treatment samples of refractory patients, suggesting that *miR-29a* may be related to paclitaxel treatment failure in BC patients. This result was in line with the literature, where *miR-29a* has been shown to be overexpressed after treating BC patients with chemotherapy [71].

In conclusion, the functional screening of paclitaxel-resistant miRNAs resulted in the finding of novel miRNAs involved in paclitaxel resistance. Our results showed that *miR-29a* and *miR-181b-1* may be good candidates for miRNA inhibition to overcome resistance to chemotherapy. Further experiments are necessary to elucidate the mechanism behind BC miRNA-mediated resistance to paclitaxel. However, we have provided further insights into potential targets to overcome the problem of resistance in BC patients.

## 4. Materials and Methods

### 4.1. Cell Culture

The BC cell lines MDA-MB-231, MDA-MB-436, MDA-MB-468, MDA-MB-453, MCF7, SKBr3, BT474, T47D, ZR751, HCC1569 and HCC1937 were used in this study (Table 1). The cell lines MCF7, ZR751, T47D, HCC1937 and HCC1569 were routinely grown in RPMI (Gibco, Thermo Fisher Scientific, Waltham, MA, USA) supplemented with 10% fetal bovine serum (FBS) (Gibco), whereas MDA-MB-453, MDA-MB-468, BT474, SKBr3 and MDA-MB-231 cells were grown in DMEM (Gibco) supplemented with 10% FBS. MDA-MB-436 cells were grown in DMEM media with 10% FBS and were supplemented with 10 μg/mL insulin.

### 4.2. Lentiviral miRNA Library and Functional Screen Optimization

A miRNA-encoding lentiviral library containing 698 human individual miRNAs (i.e., the complete miRnome (miRBase V.14)) was used in this research, as previously described [72]. Based on dose–response experiments, we used 1 µg/mL puromycin in MDA-MB-231 cells as a selectable marker of lentiviral infection. In order to optimize the lentiviral library for MDA-MB-231 cells, we first titrated the library using a GFP-expressing control virus. The GFP-positive cells were counted 48 h post-transfection (Supplementary Figure S5). Using this titer and comparing it with the reported titer of the virus in HEK293 cells, we adjusted the reported titers of the library using a correction factor.

For transfection, we used ~2.5 × 10^4^ cells/well seeded in 24-well plates. After an overnight incubation, we transfected the samples in duplicate with a lentiviral pool at a multiplicity of infection (MOI) of 1 or 5 along with polybrene (6 µg/mL). Transfected cells were incubated overnight before we carried out a media change and added puromycin. To validate the lentiviral transfection, we extracted DNA from ~10^5^ cells 72 h post-transfection and used vector-specific (pCDH) primers to amplify the PCR. In order to identify the population of miRNAs expressed by transfected cells before and after paclitaxel treatment, we used PCR amplicons to build libraries that were sequenced on an Ion 314 Chip using an Ion Torrent Personal Genome Machine (PGM) according to the manufacturer’s instructions (Life Technologies, Carlsbad, CA, USA). The generated FASTQ files were quality-controlled, adaptor-trimmed and aligned to the GRCh37 reference genome using a Bowtie2 aligner, as implemented in Galaxy server webspace (https://usegalaxy.eu/ (accessed on 2 July 2021)). Differential expression analyses of the identified miRNAs were carried out using the DESeq package implemented in R [73].

### 4.3. Drug Resistance Assays

To determine the amount of drug needed to inhibit cell growth by 50% (i.e., the GI_50_), 2 × 10^3^ cells were seeded in individual wells of a 96-well plate. The next day, different concentrations of paclitaxel were added to triplicate wells. After 72 h, MTT assays were performed by adding 10 µL of an MTT reagent (5 mg/mL) (Merck, Rahway, NJ, USA) to each well for 4 h. The media were then removed and the cells were dissolved in 100 µL dimethyl sulfoxide (Merck) to measure the absorbance at 595 nm using a Multiskan Ascent^®^ spectrophotometer (Thermo Fisher Scientific). The GI_50_ values were calculated using GraphPad Prism software (GraphPad Software v5.0).

### 4.4. Drug Resistance Adaptation Experiments

In order to model long-term drug resistance, we seeded 2 × 10^5^ cells in 6-well plates overnight before adding various concentrations of paclitaxel. Cells were left to grow for 20 days, changing the media every 3–4 days. After 20 days, the cells were washed with PBS and fixed with 4% formaldehyde (Merc) and 0.5% Toluidine Blue (Merck). Stained colonies were counted under a microscope.

### 4.5. Phenotypic Verification Using LNA miRNA Inhibitors or Lentiviral Overexpression

To confirm the resistant phenotype associated with a particular miRNA, we either inhibited the endogenous expression using locked nucleic acid (LNA) inhibitors or overexpressed the miRNA using lentiviral vector-based expression vectors.

For LNA experiments, we seeded 2 × 10^5^ cells/well in 6-well plates overnight before transfecting them with a turbofect reagent and 150 nM of the LNA inhibitors to target *miR-181b*, *miR-29a*, *miR-30c*, *miR-196a* or *miR-1295a* (Exiqon, Vedbaek, Denmark). A scrambled sequence LNA probe was used as a control. Forty-eight hours after transfection, total RNA was extracted and the miRNA expression was measured using qRT-PCR (see below).

To overexpress miRNAs, we transfected cells with either lentiviral expression vectors encoding individual miRNAs (Merck) or a negative control #1 (cat. no: NCLMIR001 (Merck)) in accordance with the manufacturer’s instructions. Briefly, ~2000 cells/well were transfected with a lentiviral vector at an MOI of 1 using a final concentration of 8 g/mL polybrene. After 72 h incubation, the media were replaced with media containing puromycin.

### 4.6. RNA Extraction and qRT-PCR

Total RNA was extracted using Tri Reagent^®^ in accordance with the manufacturer’s instructions (Sigma, Oakville, ON, Canada). Briefly, cells were resuspended in 1 mL of the Tri Reagent and incubated for 10 min at room temperature before adding 200 µL chloroform and centrifugation for 15 min at 14,000 rpm at 4 °C. The aqueous phase was removed and precipitated using 500 µL isopropanol before another centrifugation. The resulting pellet was washed with 75% ethanol and dried at room temperature for 30 min before resuspending in 30 µL RNAse-free water.

Complementary DNA (cDNA) was produced from the extracted RNA using a TaqMan MicroRNA Reverse Transcription Kit (Thermo Fisher Scientific), starting with 50 ng total RNA and using specific miRNA-RT oligonucleotides according to manufacturer’s instructions (Applied Biosystems, Waltham, MA, USA). PCRs were performed in triplicate using TaqMan-specific miRNA probes (Thermo Fisher Scientific) in an Applied Biosystems 7300 Fast Real-Time PCR System qPCR machine. Relative expression quantification was calculated using the 2^−ΔΔCt^ method, normalizing the Ct values of genes with the Ct values of housekeeping controls (RNU6b). The statistical analysis was performed using GraphPad Prism software (GraphPad Software v5.0).

### 4.7. Breast Cancer Patient Cohort

BC tissue samples (FFPE blocks) were retrospectively obtained from the Pathology Department of Onkologikoa Hospital, San Sebastián, Spain, from 42 BC patients. Samples were taken at the time of diagnosis and after the completion of the treatment schedule. Cases were selected and independently re-reviewed by two experienced breast pathologists. In this cohort, 28 patients were ER-positive and PR-positive, 4 patients were ER-positive only and 10 patients had TNBC. All patients were negative for HER2 expression. All patients were treated with either nanoparticle albumin-bound paclitaxel (nab-paclitaxel) (*n* = 5) or paclitaxel (*n* = 1) as a monotherapy or a combined therapy consisting of Adriamycin and cyclophosphamide with either nab-paclitaxel (*n* = 7) or paclitaxel (*n* = 29). The response to treatment was determined using magnetic resonance imaging and by an examination of post-treatment lesions by an expert pathologist. A complete response (CR) was determined to be those tumors not detectable by post-treatment magnetic resonance imaging in addition to an absence of tumor cells in the affected lesion. Written consent was obtained from patients for the inclusion of their samples in this study, and samples were collected in accordance with the Declaration of Helsinki and approved by local ethics committees (CEIC-E; ref. PI12015077).

## Figures and Tables

**Table 1:** Hormone receptor status of breast cancer cell lines used in this study and GI50 values from paclitaxel treatment.

Cell Lines	Receptor Status			GI_50_ (nM)
	ER	PR	HER2	
MDA-MB-231	−	−	−	0.61
MDA-MB-436	−	−	−	9.38
MDA-MB-453	−	−	+	1.27
MDA-MB-468	−	−	−	2.05
MCF7	+	+	−	24.59
BT474	+	+	+	7.77
T47D	+	+	+	240
ZR751	+	−	−	1000
SKBr3	−	+	+	1.31
HCC1937	−	−	−	37.86
HCC1569	−	−	+	6.52

ER: estrogen receptor; PR: progesterone receptor; HER2: Herceptin receptor.

**Table 2:** List of miRNAs and the number of times sequenced from clones resistant to paclitaxel.

miRNA *	Number of Sequenced Clones	miRNA	Number of Sequenced Clones
***miR-134***	27	*miR-411*	3
***miR-181b***	18	*miR-513a*	3
*miR-218*	11	*miR-616*	3
***miR-491***	12	***miR-650***	3
***miR-30c***	10	*miR-942*	3
*miR-1296*	9	*miR-let-7c*	3
*miR-527*	9	*miR-1184*	2
*miR-1281*	8	*miR-1302*	2
*miR-365a*	8	*miR-18b*	2
***miR-337***	7	*miR-362*	2
*miR-873*	7	*miR-508*	2
*miR-107*	6	*miR-512*	2
***miR-1295***	6	*miR-519*	2
***miR-329***	6	*miR-521*	2
*miR-let7b*	6	*miR-662*	2
*miR-1286*	5	*miR-1178*	1
*miR-15a*	5	*miR-1180*	1
*miR-101*	4	*miR-1182*	1
***miR-155***	4	*miR-1205*	1
***miR-196a***	4	*miR-129a*	1
*miR-199a*	4	*miR-1322*	1
***miR-29a***	4	*miR-197*	1
***miR-let-7a***	4	*miR-203*	1
*miR-128*	3	***miR-543***	1
*miR-181a*	3	*miR-548e*	1
***miR-23a***	3	*miR-614*	1
*miR-26b*	3	*miR-126*	1

* miRNAs in bold were selected for further validation.

**Table 3:** Summary of BC patients’ clinical characteristics used in this study.

Features		*n*
Immunohistochemical status	ER +	4
	ER + PR +	28
	Triple −	10
Histological grade	I	1
	II	29
	III	12
Tumor size (mm)	<30	7
	30–60	24
	>60	11
Treatment	Paclitaxel	1
	Nab-paclitaxel	5
	AC + paclitaxel	29
	AC + nab-paclitaxel	7
Response to treatment	CR	26
	Refractory	16

*n*: Number of patients; AC: Adriamycin and cyclophosphamide; CR: complete response.

## Data Availability

Data is contained within the article and Supplementary Materials.

## Supplementary Materials

The supporting information can be downloaded at: https://www.mdpi.com/article/10.3390/ijms252413630/s1.
