# Supplementary material for: Use of Gain-of-Function Screening to Identify miRNAs Involved in Paclitaxel Resistance in Triple-Negative Breast Cancer
Source: Int J Mol Sci. 2024 Dec 20;25(24):13630. doi: 10.3390/ijms252413630 (PMC11728027; doi:10.3390/ijms252413630)
Supplement: Supplementary file 1 [file ijms-25-13630-s001.zip › ijms-3326766-supplementary.pdf]

SUPPLEMENTARY FIGURE S1

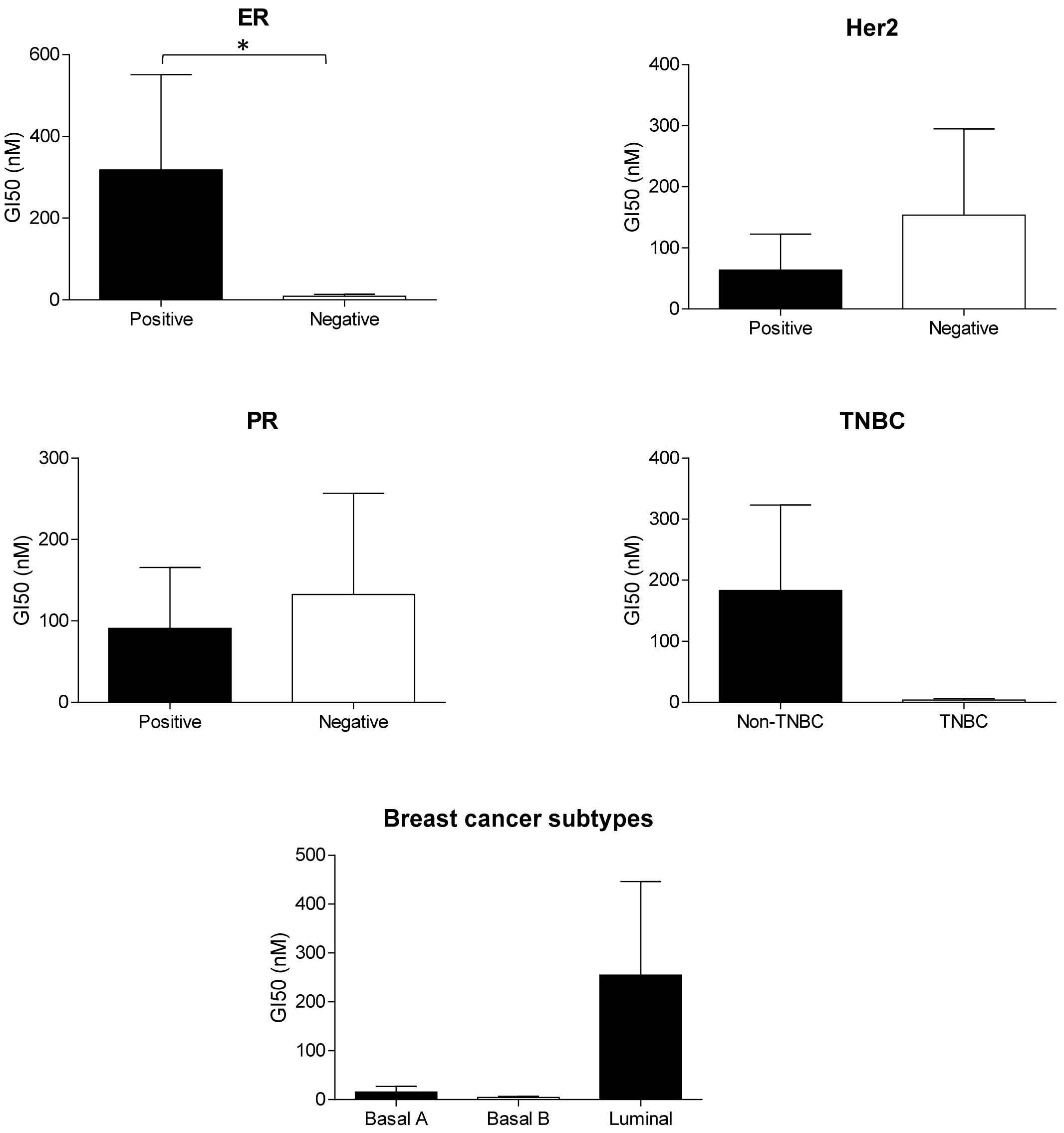

Supplementary Figure S1. Sensitivity of different breast cancer cell line subtypes to paclitaxel. GI50 values for different cell lines are represented in nM range. Cell lines are grouped depending on hormone receptor status (i.e. ER and PR) and Her2 amplification, TNBC cells are those negative for ER, PR and Her2. t-test was used for statistical analysis. In addition, cell lines are grouped depending on molecular subtypes (Basal A, Basal B and Luminal). ER negative cell lines are more sensitive to paclitaxel than ER positive cell lines ($p<0.05$). ER = Estrogen receptor; PR = Progesterone receptor, Her2= Receptor tyrosine-protein kinase erbB-2, TNBC= triple-negative breast cancer

SUPPLEMENTARY FIGURE S2

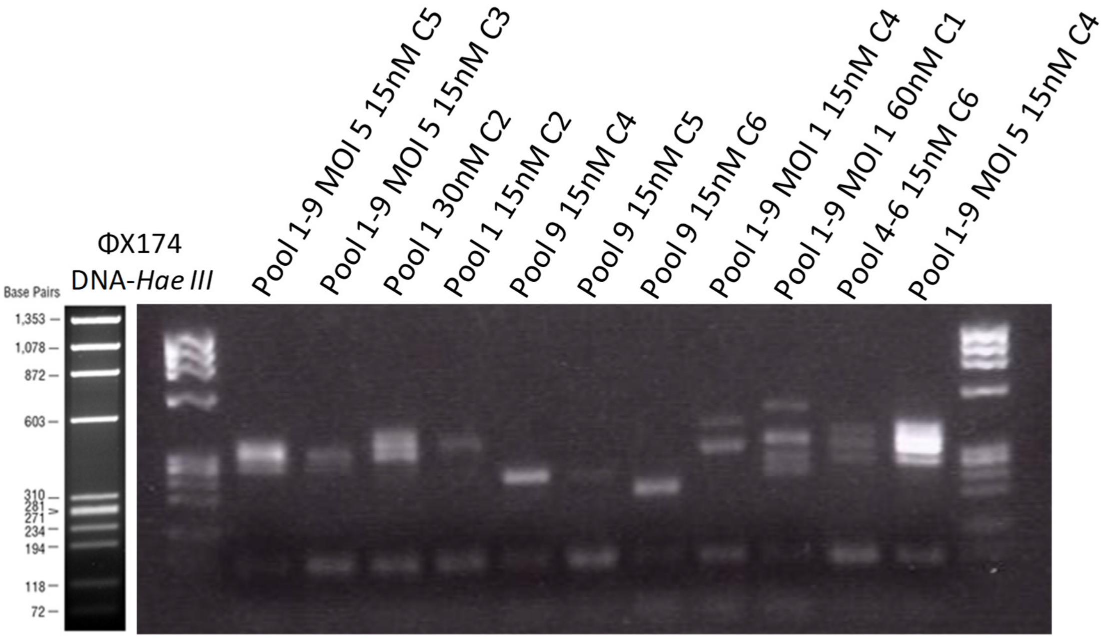

Supplementary Figure S2. Electrophoresis of paclitaxel resistant clones. PCR products from paclitaxel resistant clones were loaded into a 1.8% agarose gel. Different fragments were amplified ranging from 250 to 600bp length. The ladder used was ΦX174 DNA-*Hae III.*

SUPPLEMENTARY FIGURE S3

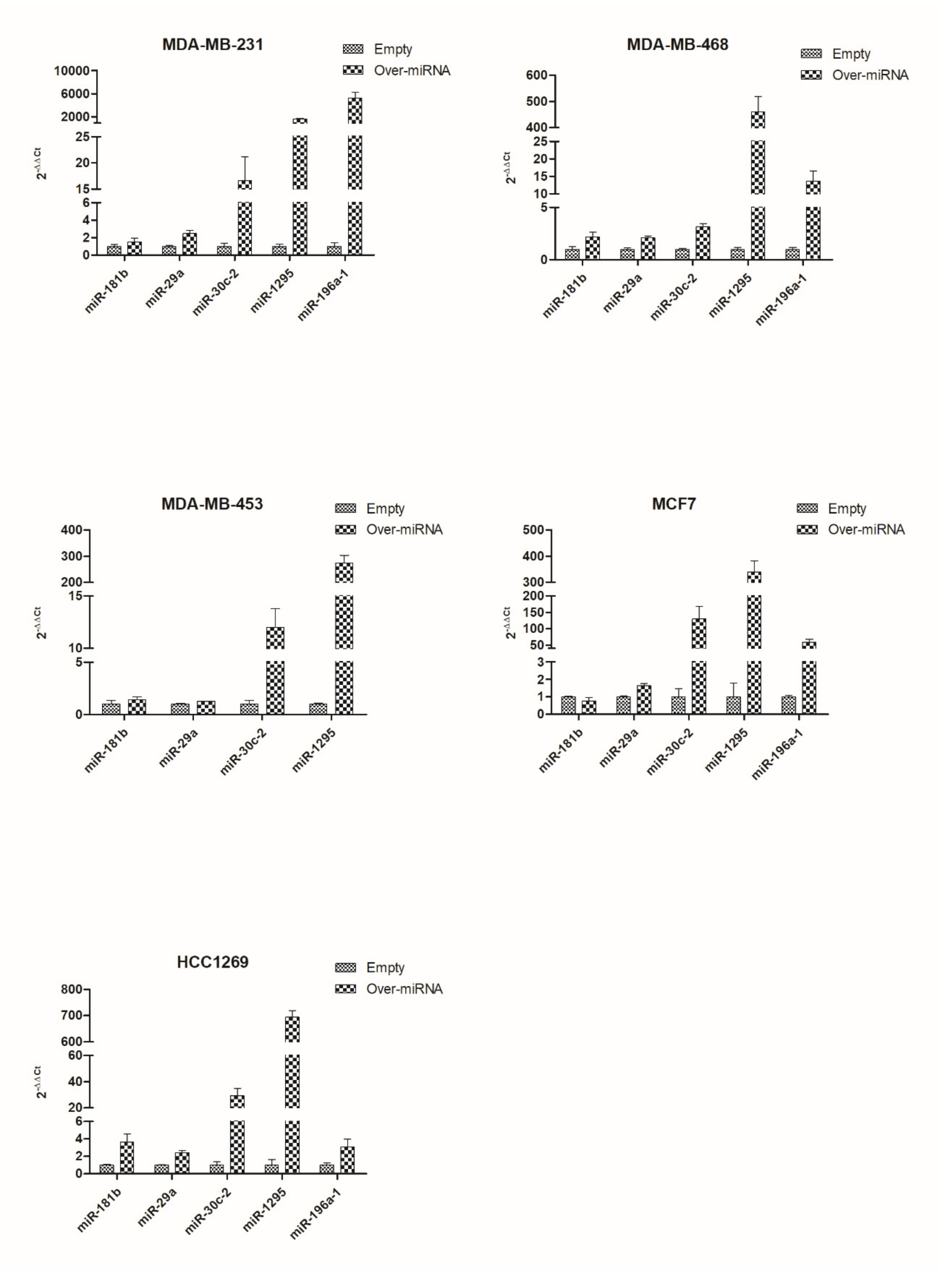

Supplementary Figure S3. qRT-PCR measurement of selected miRNAs lentiviral-transduced into various BC cell lines as shown.

SUPPLEMENTARY FIGURE S4

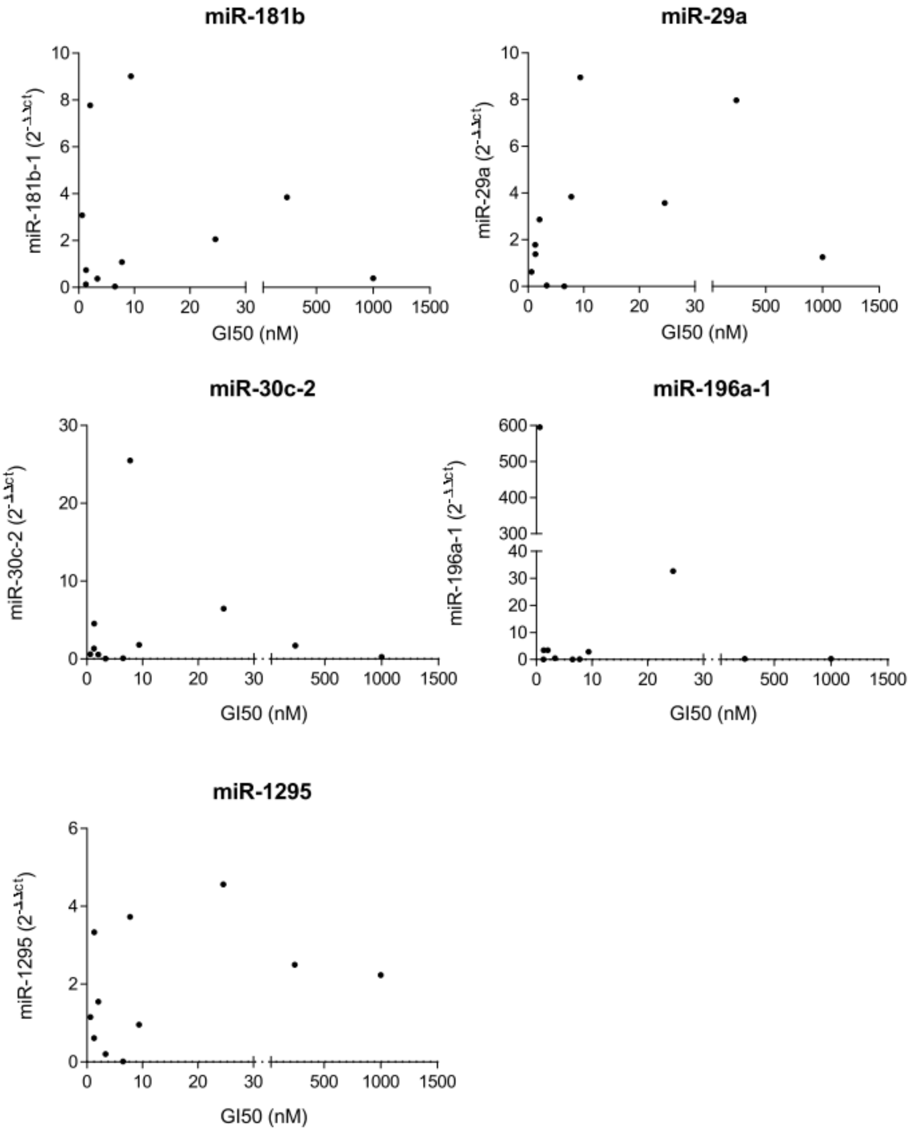

Supplementary Figure S4. Correlation between endogenous miRNA expression and GI_{50} in panel of breast cancer cell lines. The expression of *miR-181b*, *miR-29a*, *miR-30c-2*, *miR-196a-1* and *miR-1295* was measured in different cell lines (MDA-MB-231, MDA-MB-436, MDA-MB-453, MDA-MB-468, MCF7, BT474, T47D, ZR-751, SkBr3, HCC1569) by qRT-PCR using *U6b* expression as a control gene and correlated this with the values of paclitaxel GI50 in the various cell lines.

SUPPLEMENTARY FIGURE S5

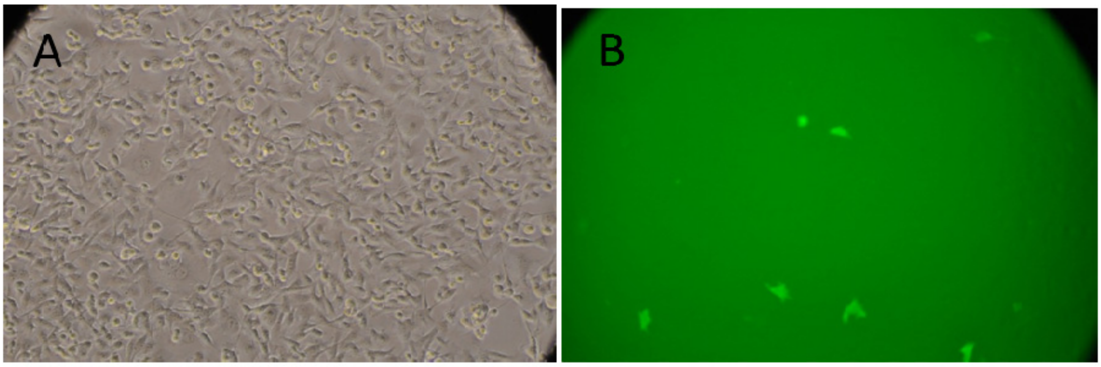

Supplementary Figure S5. MDA-MB-231 cells transduced with lentivirus (magnification 100x) (A) Brightfield image. (B) Fluorescent image showing GFP positive cells.

SUPPLEMENTARY TABLE S1

Supplementary Table S1. List of the 17 clones that resulted resistant to paclitaxel compared with parental MDA-MB-231 cell line.

# clone	Resistant clones
1	Pool 1 15nM C2
2	Pool 1 30nM C2
3	Pool 9 15nM C1
4	Pool 9 15nM C4
5	Pool 9 15nM C5
6	Pool 9 15nM C6
7	Pool 4-6 15nM C4
8	Pool 1-9 MOI 1 15nM C2
9	Pool 1-9 MOI 1 30nM C1
10	Pool 1-9 MOI 1 15nM C4
11	Pool 1-9 MOI 1 15nM C8
12	Pool 1-9 MOI 1 15nM C11
13	Pool 1-9 MOI 1 60nM C1
14	Pool 1-9 MOI 5 15nM C2
15	Pool 1-9 MOI 5 15nM C3
16	Pool 1-9 MOI 5 15nM C4
17	Pool 1-9 MOI 5 15nM C5
